# Treatment of heart failure in Dutch general practice

**DOI:** 10.1186/1471-2296-7-40

**Published:** 2006-07-05

**Authors:** Frans JM Bongers, François G Schellevis, Carel Bakx, Wil JHM van den Bosch, Jouke van der Zee

**Affiliations:** 1 NIVEL (Netherlands Institute of Health Services Research), P.O. Box 1568 3800 BN Utrecht, The Netherlands; 2 Department of General Practice and Social Medicine, University Medical Centre St Radboud Nijmegen PO Box 9101, 6500 HB Nijmegen, The Netherlands

## Abstract

**Background:**

To study the relation between the prescription rates of selected cardiovascular drugs (ACE-inhibitors and Angiotensin receptor blockers, beta-blockers, diuretics, and combinations), sociodemographic factors (age, gender and socioeconomic class) and concomitant diseases (hypertension, coronary heart disease, cerebrovascular accident, heart valve disease, atrial fibrillation, diabetes mellitus and asthma/COPD) among patients with heart failure cared for in general practice.

**Methods:**

Data from the second Dutch National Survey in General Practice, conducted mainly in 2001. In this study the data of 96 practices with a registered patient population of 374.000 were used.

Data included diagnosis made during one year by general practitioners, derived from the electronic medical records, prescriptions for medication and sociodemographic characteristics collected via a postal questionnary (response 76%)

**Results:**

A diagnosis of HF was found with 2771 patients (7.1 in 1000). Their mean age was 77.7 years, 68% was 75 years or older, 55% of the patients were women. Overall prescription rates for RAAS-I, beta-blockers and diuretics were 50%, 32%, 86%, respectively, whereas a combination of these three drugs was prescribed in 18%. Variations in prescription rates were mainly related to age and concomitant diseases.

**Conclusion:**

Prescription is not influenced by gender, to a small degree influenced by socioeconomic status and to a large degree by age and concomitant diseases.

## Background

General practitioners (GPs) play a central role in the diagnosis and management of heart failure (HF). Over half of the patients with HF are diagnosed in primary care, and one third is solely managed by the GPs [1,2]. In the last 15 years, new insights have changed the treatment of HF. In the 1970s and 1980s, physicians considered heart failure principally as a hemodynamic disorder; from the late eighties onwards they realised that it is a neurohormonal disorder [3] as well. The new concept has led to the recommendation in most guidelines [4-6] to treat patients with stable HF not only with diuretics, but also with inhibitors of the renine-angiotensin-aldosterone system (RAAS-Is) [7,8] and beta-blockers [9-11]. Currently, angiotensin-converting enzym-inhibitors (ACE-Is) are first choice among the RAAS- inhibiting drugs, but in case of side effects and adverse reactions angiotensin II receptor blockers (ARBs) are recommended as second choice [12,13]. In 1996, the Dutch College of General Practitioners issued guidelines for the diagnosis and treatment of HF. These guidelines did not include beta-blockers in the recommended medication; however, in the revised version of 2004 beta-blockers were included. In addition to these medicines digoxin [14] is still indicated in selected cases, and for patients suffering from HF with NYHA class 3 and 4 spironolactone [15] is recommended.

Recent surveys suggest that ACE-Is and beta-blockers are underprescribed in general practice [1-17].

The main aim of this study was to investigate the prescription rates of RAAS-Is, beta-blockers, diuretics, spironolactone and digoxin for patients diagnosed with HF in general practice by using a nationally representative database; these rates reflect the average prescription patterns in Dutch general practice.

We have examined the prescription rates of all patients known with HF in relation to sociodemographic (age, gender and socioeconomic class) and morbidity characteristics (specific concomitant disorders: hypertension, coronary heart disease, cerebrovascular accident, heart valve disease, atrial fibrillation, diabetes mellitus and asthma/COPD). Identification of subgroups with suboptimal treatment may guide interventions aimed at improving the quality of pharmacological treatment by GPs.

## Methods

### Design

Data were obtained from the second Dutch National Survey of General Practice (DNSGP-2), which was performed by the Netherlands Institute for Health Services Research (NIVEL) in 2001 [18]. In this survey, 195 GPs (165 GP full time equivalents) in 104 practices participated with a total practice population of 394.192 (midtime population), comprising a 2.5% sample of the Dutch population. For various reasons eight of the participating practices were excluded, leaving a midtime population of 374.000 (three practices did not deliver any morbidity data due to technical problems, the data of five practices did not meet the required quality criteria). The participating GPs were representative for Dutch GPs and practices with respect to age, gender and location in deprived areas, however, single-handed practices were underrepresented (32% instead of 44% nation wide). The patient population is representative for the Dutch population concerning age, gender, degree of urbanisation, social class and ethnic minority groups, and type of health insurance. In the Netherlands, GPs have a gatekeeper position in the health care system. All non-institutionalised patients are registered with a GP. Medical specialists are only accessible after referral by a GP. If a specialist starts treatment, in nearly all cases the GP will be responsible for the repeat prescriptions.

### Measurements

Data about age, gender and type of health care insurance (public/private) were derived from the administration of the practices. Sociodemographic data of patients were obtained by sending a questionnaire by mail to all listed patients to collect data about occupational and educational status and country of birth.

### Ethical approval

The study was carried out according to Dutch legislation on privacy. The privacy regulation of the study was approved by the Dutch Data Protection Authority. According to Dutch legislation, obtaining informed consent is not obligatory for observational studies

The overall response was 76.5%. The non-responders showed no selection with respect to age and gender, but the non-indigenous population was underrepresented in the census: 12,5 percent in the response-group versus 17,5 in the Dutch population.

To examine socioeconomic gradients the data about occupational and educational level were aggregated in three socioeconomic classes: high, medium en low. The occupational level was used as primary marker for social class. In case of unknown occupation the highest educational level was used as indicator.

Information about morbidity was derived from the electronic medical records kept by the GP. Data included health problems presented within a consultation during twelve consecutive months and diagnoses were coded using the International classification of primary care (ICPC). Also, all GP's prescriptions were extracted and coded according to the Anatomical Chemical Classification system (ATC). Patients with HF were defined on the basis of at least one contact diagnosis with ICPC code K77 during the observation year. The selected concomitant diseases were based on their respective ICPC codes in the same year. Hypertension, coronary heart disease, valve diseases and atrial fibrillation are not only important coexisting disorders but they also contribute to the development of HF and play a key role in its progression and response to therapy [19].

Prescription rates were calculated as proportions of patients with HF. We used chi-square tests to compare the effect of gender, age group, socioeconomic status and comorbidity on prescription rates.

## Results

### Patient characteristics (table 1)

**Table 1:** Number of patients with HF and prevalence rate of HF by age and sex

**Age**	**Number of patients with HF**			**Prevalence rate HF (/1000)**		
	*all*	*male*	*female*	*all*	*male*	*female*
• 0–24	5	1	4	<0.1	<0.1	<0.1
• 25–44	19	11	8	0.2	0.2	0.2
• 45–54	71	39	32	1.3	1.4	1.2
• 55–64	224	159	65	5.2	7.5	3.1
• 65–74	555	309	246	21.7	26.3	17.7
• 75 and older	1897	729	1168	91.7	96.7	85.6
• All ages	2771	1248	1523	7.4	6.7	8.1

In total 2771 patients (7.4 in 1000) were diagnosed as suffering from of heart failure: 1248 (6.7 in 1000) males and 1523 (8.1 in 1000) females. The mean age of all patients was 77,7 years (SD 10.5) ; for males it was 75.2 (SD 10.6) years, for women 79.7 years (SD 10.0); 1897 (68%) of all patients were 75 years or older. From the age of 45 onwards, every decade there was a fourfold increase in the prevalence rate of HF.

During the registration period 303 patients died; this amounted to 11% of all known patients with HF. The mean age of the deceased was 82.1 years.

### Prescription rates (table 2)

**Table 2:** Prescription rates for diuretics, RAAS-Is, beta-blockers alone or in combination*

**Medication**	**All**	**Sex**		**Below or above 75 y**		**SES**	
		Male	Female	<75 y	>=75 y	low	high
	N = 2771	1248	1523	873	1898	1235	253
***Triple treatment (%)***							
• Diuretic and RAAS-I and beta-blocker	18.0	18.4	17.7	**23.7**	**15.4**	**16.7**	**22.1**
***Two Drugs (%)***							
• Diuretic and RAAS-I	28.2	29.0	27.6	**21.7**	**31.2**	30.5	26.1
• Diuretic and beta-blocker	10.6	9.8	11.2	11.1	10.3	10.5	10.7
• RAAS-I and beta-blocker	1.3	1.1	1.4	**1.9**	**1.0**	1.3	2.4
***Monotherapy (%)***							
• Diuretic monotherapy	29.6	28.0	30.9	**24.7**	**31.2**	29.1	25.3
• RAAS-I monotherapy	3.0	3.5	2.5	**4.6**	**2.2**	2.8	3.6
• beta-blocker monotherapy	1.7	2.1	1.7	2.3	1.4	1.7	3.1

***Prescription of every drug separately (%)***							
• Diuretics	86	85	88	**83**	**88**	87	83
• RAAS-Is	50	51	48	51	49	50	53
• Betablockers	32	32	32	**40**	**29**	**31**	**39**
• Spironolactone	20	20	20	20	20	21	21
• digoxin	25	23	26	**19**	**27**	25	23

* bold figures represent statistically significant differences on the chi-square test with p < 0.05

#### Combination regimes

A combination of a diuretic with an RAAS-I and a beta-blocker (triple treatment) is considered as the basic regime for patients with HF. We investigated the various combinations of these three drugs. This *triple treatment *was used by 18% of all patients. We found statistically significant differences between the age-groups and socio-economic classes: the below-75 years group and the highest socio-economic class were prescribed more frequently the triple treatment.

Looking at *a combination of two of these three drugs*, the combination diuretics and RAAS-Is occurred in 28.2%, diuretics and beta-blockers in 10.6%, and beta-blockers and RAAS-Is in 1.3 percent. The combination diuretics-RAAS-I was seen more often in the group of 75 years and older, the combination of RAAS-I and beta-blocker more often in the group below 75 years. Diuretics as *monotherapy *were prescribed in 29.6% of all patients, RAAS-Is in 3.0% and beta-blockers in 1.7%. Here again significant differences were seen between the age groups.

#### Prescription rates for the separate drugs

One or more *diuretics *were used by 86% of all patients: in 75% of the HF patients loop diuretics were involved. RAAS-Is were prescribed in 1373 patients (50%). During the observation year 57 patients (2%) switched from an ACE-I to an ARB. *Beta-blocking *drugs were prescribed to 32% of the patients, *spironolactone *to 20%, and *digoxin *to 25%. Considering *gender*, no significant differences in prescription rates were seen for any of the medicines under investigation.

In the age group of *75 years and older *prescription rates for diuretics and digoxin were higher, but lower for beta-blockers compared to the under 75 group. Socioeconomic differences were only found for prescription of beta-blockers with a higher rate in the highest socioeconomic class.

#### Concomitant disorders

Before studying the prescription rates for concomitants disorders, we determined in which proportion the selected diseases occurred in our population of patients with HF (table 3). Overall, 30% of the patients had no comorbidity at all, 36 percent one, 23 percent two, 9 percent three and 2 percent had four or more comorbidities. Hypertension was the most common comorbidity (31%) followed by coronary heart disease (28%), diabetes mellitus (20%), asthma/COPD (20%), atrial fibrillation (14%) and CVA/TIA (8%)

**Table 3:** Prescription rates in patients with HF in relation to comorbidity*

	all	CHD	HT	CVA/TIA	AF	DM	Astma/COPD
	N = 2771	N = 769	N = 720	N = 212	N = 387	N = 551	N = 559
**Medication (%)**							
Triple	18	**30**	**27**	14	**23**	**24**	**13**
Diuretics	87	**90**	**90**	89	**90**	**90**	**92**
RAAS-Is	50	**60**	**62**	47	55	**62**	48
Beta-blockers	32	**51**	**45**	30	**40**	35	**24**
Spironolactone	20	23	21	13	21	**24**	22
digoxin	25	**21**	24	24	**64**	**29**	26

* bold figures represent statistically significant differences on the chi-square test with p < 0.05

CHD = coronary heart disease HT = hypertension AF = atrrial fibrillation DM = diabetes mellitus

Comorbidity influenced the prescription rates of the medicines under consideration. Patients with coronary heart disease, hypertension and diabetes mellitus were taking in a higher proportion nearly all drugs under study including the triple treatment. As expected, patients with atrial fibrillation used more frequently digoxin and patients with asthma or COPD less often beta-blockers. The more comorbidities, the more medicines were used (data not shown). RAAS-Is were used by 70% of the patients with three or more comorbidities.

## Discussion

This study is unique as it describes the prescription patterns for HF in an unselected general practice population in the Netherlands. In comparison with HF patients in clinical trials and in community-based studies, such a population tend to have a higher mean age [20], a higher proportion of women [21], and a greater percentage of HF with preserved left ventricular function [22,23] With a mean age of 77.7 years and a female proportion of 55%, our study population confirmed the findings for age and gender. About the percentage of patients with preserved left ventricular function we have no information.

The prevalence of 7.4 in 1000 is in line with the findings of Murphy [24] in Scotland. In most studies only the prescription rates of separate drugs were explored, in this study we investigated also the combined prescription of diuretics, RAAS-Is and beta-blockers. This triad was prescribed to approximately one out of five patients, with a significantly higher percentage in the under 75, the higher socioeconomic group and in patients with cardiovascular comorbidity, and with no differences for gender.

Considering each group of drugs separately: RAAS-Is were prescribed in about half of the cases with no significant differences for gender, age and socioeconomic status. A patient suffering from coronary heart disease, hypertension or diabetes mellitus had a chance of more than 60% to receive a RAAS-I. This proportion increased to 70% in case of three or more comorbidities in the same patient. beta-blockers were prescribed to one third of all patients with a higher proportion in the younger age-group and highest socioeconomic class. Persons with coronary heart disease had a prescription rate above 50 percent, patients with hypertension and atrial fibrillation had prescription rates above 40 percent, people suffering from asthma or COPD had a lower rate (24%).

Our results demonstrated that age and comorbidity influenced prescriptions substantially, socioeconomic class only with regard to the triple treatment, and that gender had no influence. In table 4 we summarize the prescription rates in other primary care studies and compare them with our findings. The prescription behaviour of Dutch GPs is approximately as high as in other studies; however, beta-blockers and spironolactone seem to be prescribed more often in the Netherlands than in the UK. In other studies, combination treatments of medicines were not investigated.

**Table 4:** Prescription rates in several studies

	*This study*	*Pont*^*[25]*^	*Murphy*^*[24]*^	*Key Health Statistics*^*[26]*^	*Rutten[27]*
Country	NL	NL	Scotland	UK	NL
No. of patients	2771	2493	1007	17817	103
***Medication (%)***					

• ACE-I	45	42	39	48	40
• ARB	6	9	5		6
• Beta-blocker	32	26	21	11	9
• Spironolactone	20	11	9		11
• digoxin	25	25	22	28	

### Limitations of this study

As any study of this type, this study too has its limitations. Firstly, we take the GPs' diagnosis of HF at face value, we have no independent confirmation of the diagnosis. In some studies, doubt has been raised about the validity of the diagnosis heart failure made by a GP [28,29]. However, our study aimed to study the prescription behaviour of GPs towards HF patients in primary care, so it seems justified to take the GPs' diagnosis as point of departure.

Secondly, no data about the dosages of the medicines involved are used. Thirdly, we have no information on the severity of the disease in our patient group. Fourthly, we can not differentiate between patients suffering from HF with left ventricular dysfunction and those with preserved left ventricular function.

How to judge our results? Is it acceptable that half of the patients receive RAAS-Is, one in three patient a beta-blocker and one in the five patients triple treatment?

In the IMPROVEMENT of Heart Failure Programme [1] the primary care physician's knowledge and perceptions about the management of HF were assessed. The conclusion was that knowledge of ACE-Is was high, but the physicians were less convinced about the benefits of beta-blockers. Guidelines for HF are largely based on surveys in which elderly patients and patients with multiple comorbidities are excluded. Moreover, in most studies only patients with HF and left ventricular dysfunction are included, whereas patients with preserved left ventricular function are left out. Scientific evidence about the beneficial effects of RAAS-Is and beta-blockers in patients with preserved left ventricular function is scarce [27]. In 2001, the guidelines of the Dutch Association of General practitioners did not yet recommend beta-blockers for HF.

An impression of the achievable prescription rates can be derived from Brotons [30] and Baxter [31]. Brotons et al. determined in a population of persons two years after their first myocardial infarction that the achievable standard for ACE-Is was 50%, whereas 32% were actually receiving it ; for beta-blockers these figures were 70% and 50%, respectively.

Baxter et al. determined in the setting of a geriatric outpatient department the tolerability and symptoms changes associated with the introduction of bisoprolol treatment in older patients with HF. The bisoprolol was tolerated by 69% of the 51 patients with a mean age of 78 years. When we apply these figures cautiously to our study population of patients with heart failure and hypothesize that 30% of our population had justified reasons not to use a RAAS-I, the achievable prescription rate is 70%; with the actual prescription rate of 50% there is a gap of 20%. Only persons with three or more comorbidities in our population received RAAS-Is in a proportion of 70%.

For beta-blockers we can follow a similar reasoning. Assuming that 80 percent of the patients is eligible for treatment with a beta-blocker and that 30% of the patients have justified reasons for not using it, the achievable prescription rate should be 50%. Compared with the actual rate of 32%, there is a gap of nearly 20%.

## Conclusion

Considering the observed prescription rates, the conclusion must be that, on the one hand, there is room for improvement in the treatment of patients with HF in general practice, but, on the other hand, the gap between achievable standards and actual treatment may be smaller than generally suggested. The influence of gender and socioeconomic class on prescription rates is not very marked, the influence of age and comorbidity is considerable.

Despite best practice, it may not be achievable for some patients to reach the recommended medication for various reasons, such as comorbidity, contraindications or side effects. All these reasons will occur more often in an elderly population. In the United States, 20 percent of the Medicare beneficiaries have five or more chronic conditions and 50 percent are receiving five or more medications [32]. Viewing disease-specific medication guidelines from this perspective, the question raises whether what is good for the disease is always best for the patient.

In the Netherlands, the GP has an overview of the whole medical history of a patient. Therefore, he is in the best position to translate disease guidelines into prescribing decisions for individual patients with multiple chronic conditions by weighting benefit and harm associated with multi-drug regimes. Therefore he should be supported by evidence and guidelines which are less disease-driven and more patient-driven.

## Abbreviations

ACE-I: angiotensin-converting enzym-inhibitors

ARB: angiotensin II receptor blocker

ATC: Anatomical Chemical Classification system.

DNSGP-2: second Dutch National Survey of General Practice

GP: general practitioner

HF: heart failure

ICPC: International classification of primary care

NYHA: New York Heart Association Classification

RAAS-I: renine-angiotensin-aldosterone system inhibitors

## Competing interests

The author(s) declare that they have no competing interests.

## Authors' contributions

FB was responsible for the design, for the analyses, and wrote the article

FS participated in the analyses and the writing

CB, WvdB and JvdZ critically reviewed the article.

All authors approved the final manuscript.

## Funding

The Dutch ministry of Health, Welfare and Sports mainly funded the survey.

## Pre-publication history

The pre-publication history for this paper can be accessed here:


